# Complete exon sequencing of all known Usher syndrome genes greatly improves molecular diagnosis

**DOI:** 10.1186/1750-1172-6-21

**Published:** 2011-05-11

**Authors:** Crystel Bonnet, M'hamed Grati, Sandrine Marlin, Jacqueline Levilliers, Jean-Pierre Hardelin, Marine Parodi, Magali Niasme-Grare, Diana Zelenika, Marc Délépine, Delphine Feldmann, Laurence Jonard, Aziz El-Amraoui, Dominique Weil, Bruno Delobel, Christophe Vincent, Hélène Dollfus, Marie-Madeleine Eliot, Albert David, Catherine Calais, Jacqueline Vigneron, Bettina Montaut-Verient, Dominique Bonneau, Jacques Dubin, Christel Thauvin, Alain Duvillard, Christine Francannet, Thierry Mom, Didier Lacombe, Françoise Duriez, Valérie Drouin-Garraud, Marie-Françoise Thuillier-Obstoy, Sabine Sigaudy, Anne-Marie Frances, Patrick Collignon, Georges Challe, Rémy Couderc, Mark Lathrop, José-Alain Sahel, Jean Weissenbach, Christine Petit, Françoise Denoyelle

**Affiliations:** 1 Unité de Génétique Médicale, INSERM UMRS 587, Hôpital d'Enfants Armand-Trousseau, Assistance Publique-Hôpitaux de Paris (AP-HP), Paris, France; 2 Unité de Génétique et Physiologie de l'Audition, INSERM UMRS 587, UPMC, Institut Pasteur, Paris, France; 3 Service de Biochimie et de Biologie Moléculaire, INSERM UMRS 587, Hôpital d'Enfants Armand-Trousseau, AP-HP, Paris, France; 4 Centre National de Génotypage, CEA, Evry, France; 5 Centre de Génétique, Hôpital St-Antoine, Lille, France; 6 Service ORL, Hôpital St-Antoine, Lille, France; 7 Service de Génétique médicale, Hôpital de Hautepierre, Strasbourg, France; 8 Service ORL, Hôpital de Hautepierre, Strasbourg, France; 9 Service de Génétique, Hôtel Dieu, Nantes, France; 10 Service ORL, CHU Hôtel Dieu, Nantes, France; 11 Maternité Régionale Adolphe-Pinard, Nancy, France; 12 Service ORL, Maternité Régionale Adolphe-Pinard, Nancy, France; 13 Centre de Référence des Maladies Neurogénétiques, Centre Hospitalier Universitaire d'Angers, France; 14 Service ORL, Centre Hospitalier Universitaire d'Angers, France; 15 Unité de Génétique Médicale, Hôpital, Dijon, France; 16 Service ORL, Hôpital, Dijon, France; 17 Génétique Médicale, Hôtel-Dieu, Clermont-Ferrand, France; 18 Service ORL, Hôtel-Dieu, Clermont-Ferrand, France; 19 Centre de Génétique, Hôpital Pellegrin, Bordeaux, France; 20 Service ORL, Hôpital Pellegrin, Bordeaux, France; 21 Unité de Génétique Clinique, Hôpital Charles-Nicolle, Rouen, France; 22 Service ORL Pédiatrique, Hôpital Charles-Nicolle, Rouen, France; 23 Service de Génétique Médicale, Hôpital de la Timone, Marseille, France; 24 Service de Génétique Médicale, Hôpital intercommunal de Font-Pré, Toulon La Seyne sur Mer, France; 25 Departement d'Ophtalmologie et de Médecine Interne, Hôpital de la Salpêtrière, AP-HP, France; 26 Institut de la Vision, INSERM UMRS 968, UPMC, Paris, France; 27 CEA, DSV, IG, Genoscope, CNRS-UMR 8030, UEVE, Université d'Evry, Evry, France; 28 Collège de France, Paris, France; 29 Service d'ORL et de Chirurgie Cervico-faciale, INSERM UMRS 587, Hôpital d'Enfants Armand-Trousseau, AP-HP, UPMC, Paris, France; 30 NIDCD, NIH, Bethesda, MD 20894, USA

## Abstract

**Background:**

Usher syndrome (USH) combines sensorineural deafness with blindness. It is inherited in an autosomal recessive mode. Early diagnosis is critical for adapted educational and patient management choices, and for genetic counseling. To date, nine causative genes have been identified for the three clinical subtypes (USH1, USH2 and USH3). Current diagnostic strategies make use of a genotyping microarray that is based on the previously reported mutations. The purpose of this study was to design a more accurate molecular diagnosis tool.

**Methods:**

We sequenced the 366 coding exons and flanking regions of the nine known USH genes, in 54 USH patients (27 USH1, 21 USH2 and 6 USH3).

**Results:**

Biallelic mutations were detected in 39 patients (72%) and monoallelic mutations in an additional 10 patients (18.5%). In addition to biallelic mutations in one of the USH genes, presumably pathogenic mutations in another USH gene were detected in seven patients (13%), and another patient carried monoallelic mutations in three different USH genes. Notably, none of the USH3 patients carried detectable mutations in the only known USH3 gene, whereas they all carried mutations in USH2 genes. Most importantly, the currently used microarray would have detected only 30 of the 81 different mutations that we found, of which 39 (48%) were novel.

**Conclusions:**

Based on these results, complete exon sequencing of the currently known USH genes stands as a definite improvement for molecular diagnosis of this disease, which is of utmost importance in the perspective of gene therapy.

## Background

Usher syndrome (USH, MIM 276900, MIM 276905, MIM 605472) combines sensorineural hearing impairment with retinitis pigmentosa [1]. In addition, vestibular dysfunction can be observed in some patients. USH occurs in ~1/20 000 individuals, and represents 50% of all monogenic deaf-blindness cases. Three clinical subtypes can be distinguished. USH type I (USH1) is characterized by severe to profound congenital hearing impairment, prepubertal onset of retinitis pigmentosa, and vestibular arreflexia. USH type II (USH2) combines congenital moderate to severe hearing impairment, onset of retinitis pigmentosa in the first or second decade of life, and absence of vestibular dysfunction. Finally, USH type III (USH3) patients present with congenital or early onset progressive hearing impairment, variable age of onset and severity of retinitis pigmentosa, and variable vestibular dysfunction. USH is inherited in the autosomal recessive mode, and is genetically heterogeneous. To date, nine causative genes have been identified. Mutations in *MYO7A *[2], *USH1C *[3,4], *CDH23 *[5,6], *PCDH15 *[7,8] and *USH1G *[9] cause USH1, mutations in *USH2A *[10], *VLGR1 *[11] and *WHRN *[12] cause USH2, and mutations in *USH3A *[13] cause USH3. Mutations in *MYO7A *[14-16], *USH1C *[17,18], *CDH23 *[6], *PCDH15 *[17] and *WHRN *[19] have also been reported in patients affected by hearing impairment only, while *USH2A *is also involved in isolated retinitis pigmentosa [20].

The USH1 genes encode the actin-based motor protein myosin VIIa (USH1B), two Ca^2+^-dependent transmembrane adhesion proteins, cadherin-23 (USH1D) and protocadherin-15 (USH1F), the PDZ domain-containing submembrane protein harmonin (USH1C), and the scaffold protein sans that contains ankyrin repeats and a sterile alpha motif domain (USH1G). The USH2 genes encode two large transmembrane proteins, usherin (USH2A) and VLGR1 (very large G protein-coupled receptor, USH2C), and the PDZ domain-containing submembrane protein whirlin (USH2D). Finally, *USH3A *encodes the four-transmembrane-domain protein clarin-1. Each USH gene encodes several protein isoforms, except *MYO7A *and *USH1G*.

Absence of an early diagnosis of USH is devastating. In USH1 patients, sign language becomes a less and less efficient mode of communication as the visual defect progresses, and ultimately, the patients may become unable to communicate except by tactile exchanges. As a result of an early diagnosis of USH1, early bilateral cochlear implantation allowing the development of an oral mode of communication and early physical therapy for vestibular disorders are strongly recommended. The early diagnosis is also critical for genetic counseling, educational orientation and therapeutic management, which may include retinal gene therapy in the future [21,22]. So far, a comprehensive molecular diagnosis of USH has been hampered both by the genetic heterogeneity of the disease and the large number of exons for six out of the nine known USH genes. The five USH1, three USH2, and one USH3 genes are collectively composed of 183, 173, and five coding exons, respectively [23].

Cremers and collaborators have developed a genotyping microarray for USH, based on the arrayed primer extension (APEX) method. This approach, in a first version, included the analysis of 298 USH-associated sequence variants located in eight genes: *MYO7A*, *USH1C*, *CDH23*, *PCDH15*, *USH1G*, *USH2A*, *VLGR1 *and *USH3A *[24]. The mutations detected by the array subsequently increased, and currently include 612 previously identified disease-associated variants in the nine known USH genes [25]. The selected variants were prevalent in the following European countries: Belgium, Denmark, UK, Germany, Italy, Spain, Switzerland and Netherlands, and in the USA. The authors could prove that the chip, with >98% accuracy, is an adaptable and affordable mutation screening tool. However, the efficiency of the chip was both dependent on the USH subtype examined and the studied population, ranging from 30% in the USA to 80% in Denmark in USH1 cases [24]. Recently, Jaijo et al., using an intermediate genotyping microarray (429 reported mutations), found mutations in only 34% of the patients tested [26], which is indicative of a large number of private mutations. Therefore, improvement of the molecular diagnosis is needed.

Alternative strategies include direct sequencing of USH gene coding exons [27-30]. To determine the most efficient strategy, some critical information is, however, still lacking. Is the clinically diagnosed USH subtype a reliable indication of the causative gene? What is the frequency of digenic/oligogenic inheritance in this disease? Such a mode of inheritance is suggested by the colocalization and direct *in vitro *interactions of the USH1 proteins [31-39], and of the USH2 proteins [40,41]. In a few USH1 patients, digenic inheritance involving *PCDH15 *and *CDH23 *has indeed been reported [42]. To address these issues, we undertook a large-scale mutation screening of all currently known USH genes in a cohort of 54 USH patients.

## Subjects and Methods

### Subjects

Fifty-four unrelated Caucasian patients including five patients originating from Maghreb were included in the study. Most patients were referred to Armand-Trousseau Children's Hospital in Paris, and other patients were referred to genetic consultations throughout France. All patients were tested by audiograms and electroretinogram. Auditory function was assessed by otoscopy, tympanometry, standard pure tone audiometry, and recording of auditory brainstem responses and otoacoustic emissions. The cochlear origin of the hearing impairment was confirmed by auditory brainstem responses, and by the absence of otoacoustic emissions. USH was diagnosed on the basis of simultaneous occurrence of sensorineural deafness and retinal degeneration. Scrutiny of the time of onset, evolution and severity of the hearing impairment, and quality of vestibular responses enabled to assign the patients to one of the three clinical types of the disease [43]. Patients were considered as USH3 when their hearing impairment had been detected in adulthood and showed clear progressiveness. For these patients, vestibular function determined by caloric tests was normal. Parents of most of the patients were available for the study, and had normal hearing. This study was approved by the local ethics committee, and written consent for genetic testing was obtained from adult probands or parents of minor patients.

### PCR amplification and sequencing

Genomic DNA was extracted from peripheral blood using standard procedures. The coding exons and flanking intronic sequences of all nine USH genes were amplified and sequenced using forward and reverse primers (primer sequences and conditions available upon request). We also searched for the previously reported 684 kb deletion in *PCDH15 *using the reported primers [44]. Sequences were run on ABI 3100 DNA analyzer, and assembled using ABI Prism Seqscape 2.1 to Genbank reference sequences [45].

### Control DNAs

The genomic DNAs from 153 unaffected Caucasian control individuals were sequenced (306 control alleles). For the mutations possibly involved in oligogenic inheritance, DNAs from 333 healthy unrelated Caucasian individuals were used as controls. For the mutations present in patients originating from Maghreb, the DNAs from 95 Moroccan and 91 Algerian healthy unrelated individuals were used as controls.

### In silico analysis of sequence variants

The SIFT (Sorting Intolerant from Tolerant) [46] and Polyphen [47] software programs were used to predict the influence of any amino acid substitution on the protein structure and function. NetGene2 [48] and "Splice site prediction by neural network" [49] interfaces were used to predict the influence of nucleic acid substitutions on splice donor and acceptor sites. Presence of Exonic Splicing Enhancers (ESE) was detected using ESE Finder [50].

### Segregation analysis

Segregation of all sequence variants identified in the patients was studied by sequencing the corresponding DNA fragments in the parents and other relatives. In all patients carrying two distinct mutations in a given USH gene, biallelic transmission was confirmed by the segregation analysis.

### Mutation nomenclature

The mutation nomenclature complies with the mutation nomenclature correction tool Mutalyzer [51] according to the HGVS Guidelines & Recommendations [52]. The +1 position in mutation numbering corresponds to the A of the ATG initiation codon.

### Protein Accession numbers

*MYO7A*, [Swiss-Prot:Q13402]; *USH1C*, [Swiss-Prot:Q7RTU8]; *CDH23*, [Swiss-Prot:Q9H251]; *PCDH15-CD1*, [Swiss-Prot:Q96QU1]; *PCDH15-CD2*, [NCBI-RefSeq:NP_001136241.1]; *PCDH15-CD3*, [Swiss-Prot:C9J4F3]; *USH1G*, [Swiss-Prot:Q495M9]; *USH2A*, [Swiss-Prot:075445]; *VLGR1*, [Swiss-Prot:Q8WXG9]; *WHRN*, [Swiss-Prot:Q9P202]; *USH3A*, [Swiss-Prot:P58418] and [Swiss-Prot:P58418-1] for "a" and "c" variants, respectively.

## Results

### Mutation analysis: high prevalence of novel mutations

We analyzed the nine USH genes in a cohort of 54 French patients, of whom 27 were affected by USH1, 21 by USH2, and six by USH3. From the patient and parent questionnaires, consanguinity was established for nine families (see Table 1). Sequencing of the coding and non coding exons of all currently known USH genes was carried out in every patient. Screening for predicted causative missense and splice site mutations was performed using prediction software programs. Amino acid substitutions were considered likely to be pathogenic missense mutations when predicted possibly or probably deleterious by Polyphen software and not-tolerated by the SIFT program. Nucleotide variations were considered likely to be splice site mutations when predicted highly confident donor or acceptor site mutations by Netgene2 and "Splice site prediction by neural network" programs. These sequence variants were ultimately classified as presumably pathogenic mutations only if the affected amino acid residues were evolutionarily conserved (Additional file 1 Figures S1 to S3) and/or these variants were not found in the control individuals (see Subjects and Methods).

**Table 1:** Genotypes of USH patients

Genes			*MYO7A*	*USH1C*	*CDH23*	*PCDH15*	*USH1G*	*USH2A*	*VLGR1*	*WHRN*	*USH3A*
**Patient**		**USH type**									

**U37**		**I**	[p.R666X] + [p.E1917X]								

**U57**		**I**	**[p.C1198X]**+ [p.R1240Q]								

**P0485**		**I**	[p.Q1798X] + [p.E1917X]								

**U14**	**C**	**I**	[p.R972X] + [p.R972X]								

**U9**	**C**	**I**	**[p.K164X] **+ **[p.K164X]**								

**U36**		**I**	[p.R2024X] + [p.G519D]		[p.R1060W]						

**U20**		**I**	[p.R669X] + [p.R1883Q]								

**P0505**		**I**	[p.Q1798X] + [p.A2009fsX32]								

**S1556**	**C**	**I**	**[p.H133fsX7] **+ **[p.H133fsX7]**								

**S1295**	**C**	**I**	**[p.Y1302fsX97] **+ **[p.Y1302sX97]**					**[p.G1301V]**	**[p.Q5459H]**		

**P0504**		**I**	[p.D75fsX31] + [p.R1240Q]	**[p.R357W]**							

**U45**		**I**	[p.D75fsX31] + [p.T165M]								

**P0411**	**C**	**I**	[c.2283-1G>T] + [c.2283-1G>T]						**[p.D4707Y]**		

**P0070**		**I**	[p.G163R] + [p.A198T]								

**P0052**		**I**	**[c.1690+1G>A] **+ [p.F1963del]								

**U3**		**I**	[p.L2186P]				**[p.L16V]**	**[p.C3307W]**			

**DID**	**C**	**I**		[p.R80fsX69] + [p.R80fsX69]	**[p.R3043W]**						

**U47**		**I**		[p.R80fsX69] + [p.R103H]							

**P0469**		**I**			**[p.E2135fsX3] **+ [c.6050-9G>A]						

**S1212**		**I**			**[p.R1379P] **+ **[p.D2639G]**						

**U38**		**I**				[p.R991X] + [p.R991X]					

**S1530**		**I**				**[p.R1273S]**					

**P0257**		**I**					[p.W38X]				

**S1273**		**I**					**[p.D29fsX29] + [p.D29fsX29]**				

**U46**		**I**									

**U50**		**I**									

**S1823**	**C**	**I**									

											

**P0486**		**II**	[p.A457V] + [p.K269del]								

**U6**		**II**						[p.E3562X] + [p.E767fsX21]			

**U24**		**II**	**[p.P1220L]**					[p.S1307X] + [p.C536R]			

**U48**		**II**						[p.W3955X] + **[p.R2509fsX19]**			

**P0483**		**II**						[p.E1492X] + [p.T3571M]			

**P0418**		**II**	**[p.K268R]**					[p.S5030X]			

**U56**	**C**	**II**						**[p.T2991fsX61] **+ **[p.T2991fsX61]**			

**U42**		**II**						[p.E767fsX21] + **[p.Y4128fsX24]**			

**P0449**		**II**						[p.E767fsX21] + **[p.C575Y]**			

**P0493**		**II**						[p.H308fsX16] + [p.T4809I]			

**P0432**		**II**			**[p.R1189W]**			**[p.M1344fsX42]**			

**U51**		**II**						[p.V218E] + [p.R317R]			

**P0511**		**II**						[p.T3571M] + [p.T352I]			

**U49**		**II**							**[p.E4321X] **+ **[p.Q753fsX8]**		

**P0473**		**II**							**[p.P522fsX8] **+ **[p.M5890fsX10]**	**[p.S11R]**	

**U58**		**II**							**[p.F112fsX29] **+ **[p.H3399P]**		

**P0463**		**II**							**[p.E4186fsX17]**		

**U10**		**II**									

**U53**		**II**								**[p.P246fsX13] **+ **[p.P246fsX13]**	

**U19**	**C**	**II**			[p.H755Y]						

**P0426**		**II**									

											

**U21**		**III**						**[p.Y1730fsX6**] + **[c.10586-1G>C]**			

**U30**		**III**						[p.E767fsX21] + [p.R303H]			

**S1226**		**III**						[p.G2752R] + [c.5776+1G>A]			

**P0239**		**III**							**[p.N4885S]**		

**P0484**		**III**							**[p.D1944N]**		

**P0069**		**III**								**[p.R379W]**	

Novel mutations are in bold. C (2^nd ^column) denotes consanguinity.

**Figure 1:**
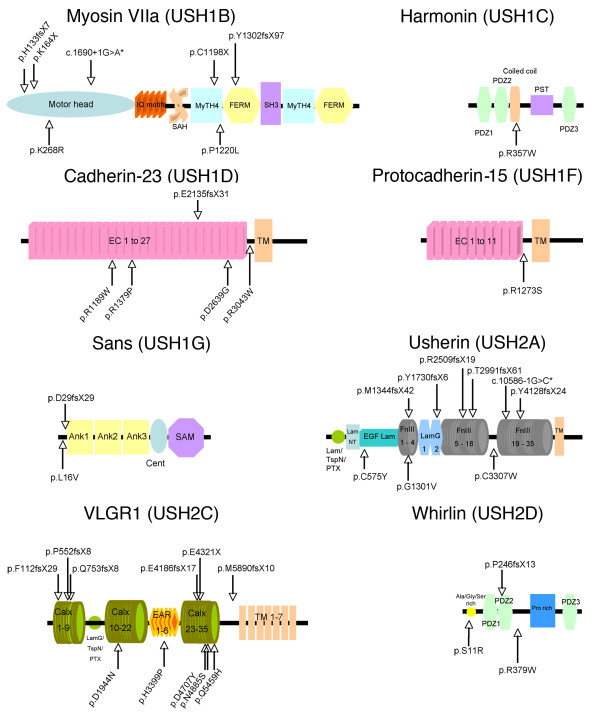
**Schematic representation of USH1 and USH2 proteins and localization of the novel, presumably pathogenic mutations**. The long isoform of each USH protein is shown. *Splice site mutations. Abbreviations: *IQ motifs*, isoleucine-glutamine motifs; *SAH*, stable single α-helix; *MyTH4*, myosin tail homology 4; *FERM*, band 4.1-ezrin-radixin-moesin; *PDZ*, PSD95, discs large, ZO-1; *PST*, proline-serine-threonine-rich region; *EC*, extracellular cadherin; *TM*, transmembrane domain; *Ank*, ankyrin domains; *cent*, central region; *SAM*, sterile alpha motif; *LamG*, laminin G; *LamG/TspN/PTX*, N-terminal thrombospondin/pentaxin/laminin G-like domain; *LamNT*, laminin N-terminal; *EGF Lam*, laminin-type EGF-like; *FnIII*, fibronectin type III; *VLGR1*, very large G protein-coupled receptor 1; *Calx*, Ca^2+^-binding calcium exchanger β; *EAR*, Epilepsy Associated Repeats; *Ala/Gly/Ser rich*, alanine, glycine, and serine rich region; *Pro rich*, proline rich region.

A total of 81 distinct, presumably pathogenic mutations were detected, specifically, 16 nonsense mutations, five nucleotide duplications, 17 frame-shifting deletions, seven splicing defect-causing mutations, 34 missense mutations, and one isocoding variation. Thirty-nine (48%) of these mutations, i.e. 27% to 100% of the mutations found in each USH gene, had not been previously reported (Tables 2, 3 and 4, Figure 1). In addition, 103 amino acid substitutions were classified as presumably nonpathogenic sequence variants, including 33 new variants and six variants that had previously been reported as pathogenic mutations (Table 5). Numerous, presumably neutral, isocoding and intronic variants were also observed (listed in Additional file 2, Table S1).

**Table 2:** Pathogenic DNA variants

Gene	Nucleotide change	Exon	Amino acid change	Frequency in USH alleles (×/108)	Frequency in control alleles	Patient origin & reference
***MYO7A***						

	223delG	4	D75fsX31	2		Australia, Italy, France [78]
	**397dupC**	5	**H133fsX7**	2		This study
	**490A>T**	6	**K164X**	2		This study
	592G>A	6	A198T + splice defect	1	0/306	Algeria [27]
	1556G>A	14	G519D/splice defect	1	0/306	USA, France [63]
	**1690+1G>A**	14	**Splice defect**	1		This study
	1996C>T	17	R666X	1		Great Britain, Denmark [62]
	2005C>T	17	R669X	1		USA [24]
	2283-1G>T	20	Splice defect	2		Algeria [27]
	2914C>T	24	R972X	2		Pakistan [79]
	**3594C>A**	28	**C1198X**	1		This study
	**3904delT**	30	**Y1302fsX97**	2		This study
	5392C>T	39	Q1798X	2		Denmark, German, Great Britain/France [62]
	5749G>T	42	E1917X	2		unknown [80]
	6025delG	44	A2009fsX32	1		Spain [63]
	6070C>T	45	R2024X	1		unknown [80]
						

***USH1C***						

	238_239dupC	3	R80fsX69	3		Pakistan, Europe, Guinea [4]
						

***CDH23***						

	6050-9G>A	46	Splice defect	1		Germany [54]
	**6404_6405delAG**	47	**E2135fsX31**	1		This study
						

***PCDH15***						

	2971C>T	22	R991X	2		France [27]
						

***USH1G***						

	**84dupC**	1	**D29fsX29**	2		This study
	113G>A	1	W38X	1		USA [58]
						

***USH2A***						

	920_923dupGCCA	6	H308fsX16	1		Denmark [81]
	2299delG	13	E767fsX21	4		Europe, USA, Africa, China [10]
	3920C>G	18	S1307X	1		France [82]
	**4030_4037delATGGCTGG**	18	**M1344fsX42**	1		This study
	4474G>T	21	E1492X	1		Spain [83]
	**5189_5199delATATGTTTCAT**	26	**Y1730fsX6**	1		This study
	5776+1G>A	28	Splice defect	1		Norway [28]
	**7522delT**	40	**R2509fsX19**	1		This study
	**8970_8971delCA**	45	**T2991fsX61**	2		This study
	**10586-1G>C**	54	**Splice defect**	1		This study
	10684G>T	54	E3562X	1		Denmark, Norway [28]
	11864G>A	61	W3955X	1		Netherlands [84]
	**12381_12382delCT**	63	**Y4128fsX24**	1		This study
	15089C>A	70	S5030X	1		France [66]
						

***VLGR1***						

	**333_334delTT**	3	**F112fsX29**	1		This study
	**1563dupT**	9	**P522fsX8**	1		This study
	**2258_2270delAAGTGCTGAAATC**	12	**Q753fsX8**	1		This study
	**12552_12553delGG**	62	**E4186fsX17**	1		This study
	**12961G>T**	64	**E4321X**	1		This study
	**17668_17669delAT**	82	**M5890fsX10**	1		This study
						

***WHRN***						

	**737delC**	2	**P246fsX13**	2		This study

Novel mutations are in bold.

**Table 3:** Presumably pathogenic DNA variants

Gene	Nucleotide change	Exon	Amino acid change	Protein domain	Frequency in USH alleles (×/108)	Frequency in control alleles	Patient origin & reference
***MYO7A***							

	487G>C	6	G163R	Motor head	1	0/306	Algeria [27]
	494C>T	6	T165M	Motor head	1	0/306	Great Britain, France [58]
	**803A>G**	8	**K268R**	Motor head	1	0/306	This study
	805_807delAAG	8	K269del	Motor head	1	0/306	Italy, France [63]
	1370C>T	13	A457V	Motor head	1	0/306	Ireland, France [63]
	**3659C>T**	29	**P1220L**	MyTH4 (1)	1	0/666	This study
	3719G>A	29	R1240Q	MyTH4 (1)	2	0/306	Denmark, Great Britain/France [62]
	5648G>A	41	R1883Q	MyTH4 (2)	1	0/306	USA [58]
	5887_5889delTTC	43	F1963del	FERM (2)	1		Europe, USA [24]
	6657T>C	48	L2186P	FERM (2)	1	0/666	France [85]
							

***USH1C***							

	308G>A	4	R103H	PDZ1	1	0/306	France [27]
	**1069C>T**	13	**R357W**	Coiled-coil	1	0/498	This study
							

***CDH23***							

	2263C>T	20	H755Y	cd7	1	0/306	USA [56]
	3178C>T	26	R1060W	cd10	1	0/626	Europe [55]
	**3565C>T**	29	**R1189W**	cd11	1	0/306	This study
	**4136G>C**	33	**R1379P**	cd13	1	0/306	This study
	**7916A>G**	55	**D2639G**	cd25	1	0/306	This study
	**9127C>T**	62	**R3043W**	adjacent to TM (extracellular)	1	0/490	This study
							

***PCDH15***							

	**3817C>A**	29	**R1273S**	cd11	1	0/306	This study
							

***USH1G***							

	**46C>G**	1	**L16V**		1	0/666	This study
							

***USH2A***							

	653T>A	4	V218E	Nter laminin	1	0/306	Great Britain [86]
	908G>A	6	R303H	Nter laminin	1	0/306	USA [87]
	949C>A	6	R317R	Nter laminin	1	0/306	Netherlands [60]
	1055C>T	6	T352I	Nter laminin	1	0/306	Norway [28]
	1606T>C	9	C536R	1^st ^laminin EGF-like	1	0/306	Denmark [81]
	**1724G>A**	10	**C575Y**	2^nd ^laminin EGF-like	1	0/306	This study
	**3902G>T**	18	**G1301V**	14^th ^FnIII	1	0/484	This study
	8254G>A	42	G2752R	3^rd ^laminin EGF-like	1	0/306	Japan [88]
	**9921T>G**	50	**C3307W**	18^th^-19^th ^FnIII	1	0/482	This study
	10712C>T	54	T3571M	20^th ^FnIII	2	0/306	Spain [89]
	14426C>T	66	T4809I	33^rd ^FnIII	1	0/306	Canada [90]
							

***VLGR1***							

	**5830G>A**	28	**D1944N**	13^th ^-14^th ^β-Calx	1	0/306	This study
	**10196A>C**	49	**H3399P**	4^th ^EAR	1	0/306	This study
	**14119G>T**	70	**D4707Y**	32^nd ^β-Calx	1	0/446	This study
	**14654A>G**	71	**N4885S**	32^nd ^-33^rd ^β-Calx	1	0/486	This study
	**16377G>T**	77	**Q5459H**	35^th ^β-Calx	1	0/402	This study
							

***WHRN***							

	**33C>G**	1	**S11R**	A/G/S rich region	1	0/494	This study
	**1135C>T**	4	**R379W**	PDZ2	1	0/306	This study

Novel mutations are in bold.

**Table 4:** Distribution of the pathogenic and presumably pathogenic mutations

	Pathogenic and presumably pathogenic mutations (Novel mutations)
*MYO7A*	26 (7)
*USH1C*	3 (1)
*CDH23*	8 (5)
*PCDH15*	2 (1)
*USH1G*	3 (2)
*USH2A*	25 (9)
*VLGR1*	11 (11)
*WHRN*	3 (3)
*USH3A*	0

**Table 5:** Presumably neutral missense variants

Gene	Nucleotide change	Exon	Amino acid change	Frequency in USH alleles (×/108)	Frequency in control alleles	References
***MYO7A***						

	47T>C	3	L16S	>10		[58]
	905G>A	9	R302H	2	1/494	[78]
	4996A>T	36	S1666C	>10		U39226
	5156A>G	37	Y1719C*	3	2/306	[91]
	5860C>A	43	L1954I	>10		U39226
						

***USH1C***						

	**2192G>A**	21	**R731Q**	1		This study
	2457G>C	24	E819D	>10		[92]
						

***CDH23***						

	7C>T	1	R3C	>10		[55]
	1469G>C	14	G490A	>10		[55]
	1487G>A	14	S496N	>10		[55]
	3625A>G	30	T1209A*	1	5/486	[55]
	3664G>A	30	A1222T	4		[55]
	4051G>A	31	D1351N	>10		[55]
	4310G>A	34	R1437Q	6		[55]
	4723A>G	37	T1575A	>10		[55]
	4858G>A	38	V1620M	1	2/306	[55]
	5023G>A	38	V1675I	>10		[55]
	5411G>A	41	R1804Q	>10		[55]
	5418C>G	41	D1806E	2		[93]
	**5692G>A**	42	**A1898T**	1	0/306	This study
	5996C>G	45	T1999S	>10		[55]
	6130G>A	46	E2044K	>10		[55]
	6197G>A	46	R2066Q	1	0/306	[55]
	**6329C>T**	47	**A2110V**	1		This study
	**6596T>A**	47	**I2199N**	1	0/306	This study
	**6809G>A**	48	**R2270H**	1		This study
	6847G>A	49	V2283I	6		[55]
	**6869C>T**	49	**T2290M**	1	0/306	This study
	7073G>A	50	R2358Q	>10		[55]
	7139C>T	50	P2380L	>10		[55]
	7762G>C	54	E2588Q	1	1/306	[55]
	**9049G>A**	61	**D3017N**	1		This study
	9373T>C	65	F3125L	1	7/306	[56]
	**9949G>A**	69	**A3317T**	1	1/306	This study
						

***PCDH15***						

	55T>G	2	S19A	>10		[94]
	**1039C>T**	10	**L347F**	1	3/666	This study
	**1138G>A**	11	**G380S**	>10		This study
	1304A>C	11	D435A	>10		AL834134
	1910A>G	15	N637S	2		[92]
	2786G>A	21	R929Q	>10		AL834134
	**4850A>G**	34**^§^**	**N1617S**	2		This study
	**4853A>C**	36**^§^**	**E1618A**	>10		This study
	**4982A>C**	37**^§^**	**Q1661P**	>10		This study
						

***USH2A***						

	373G>A	2	A125T	>10		[95]
	1434G>C	8	E478D	3		[95]
	1663C>G	10	L555V*	1	0/306	[96]
	1931A>T	11	D644V	>10		[95]
	4457G>A	21	R1486K	>10		AF055580
	4994T>C	25	I1665T	>10		[89]
	6317T>C	32	I2106T	>10		[89]
	6506T>C	34	I2169T	>10		[89]
	6713A>C	35	E2238A	6	5/306	[89]
	6875G>A	36	R2292H	4		[28]
	8624G>A	43	R2875Q	4		[89]
	8656C>T	43	L2886F	4		[89]
	**9008T>C**	45	**V3003A**	1		This study
	9262G>A	47	E3088K*	1	3/306	[28]
	9296A>G	47	N3099S	4		[89]
	9343A>G	47	T3115A	3	5/306	[28]
	9430G>A	48	D3144N	4		[89]
	9595A>G	49	N3199D	6		[28]
	10232A>C	52	E3411A	>10		[89]
	11504C>T	59	T3835I	>10		[28]
	11602A>G	60	M3868V	>10		[89]
	11677C>A	60	P3893T*	1	1/306	[28]
	15091C>T	70	R5031W	2	2/306	[28]
	15377T>C	71	I5126T*	3	2/306	[87]
						

***VLGR1***						

	**365C>T**	4	**S122L**	>10		This study
	**P194H**	6	**P194H**	1	5/468	This study
	**1033C>A**	7	**Q345K**	1		This study
	**2261T>C**	12	**V754A**	1	0/306	This study
	**3289G>A**	17	**G1097S**	1	3/478	This study
	**3482C>G**	19	**S1161C**	1	0/306	This study
	**4939A>G**	23	**I1647V**	>10		This study
	5780C>T	28	T1927M	>10		[11]
	5851G>A	28	V1951I	>10		[11]
	5953A>G	28	N1985D	>10		[11]
	5960C>T	28	P1987L	>10		[11]
	6012G>T	28	L2004F	>10		[11]
	6695A>G	30	Y2232C	>10		[11]
	7034A>G	32	N2345S	>10		[11]
	**7582C>T**	33	**P2528S**	1	1/306	This study
	7751A>G	33	N2584S	>10		[97]
	8291C>T	36	S2764L	6		[11]
	8407G>A	37	A2803T	4		[11]
	**9280G>A**	43	**V3094I**	>10		This study
	9743G>A	45	G3248D	>10		[11]
	9650C>T	45	A3217V	2		[11]
	10411G>A	49	E3471K	>10		[97]
	**10429G>T**	50	**D3477Y**	1		This study
	**10490A>G**	50	**Q3497R**	1		This study
	**10577T>C**	51	**M3526T**	3		This study
	**10936T>C**	52	**S3646P**	3		This study
	**11599G>A**	56	**E3867K**	>10		This study
	**12269C>A**	59	**T4090N**	2		This study
	**14029T>C**	69	**F4677L**	1	2/478	This study
	**14905T>C**	73	**W4969R**	2		This study
	**17626G>A**	82	**V5876I**	>10		This study
	**18475A>G**	88	**M6159V**	2		This study
						

***WHRN***						

	229A>T	1	T77S	1	1/468	[98]
	**979C>A**	4	**L327I**	1		This study
	1318G>A	6	A440T	>10		[99]
	**1838T>C**	9	**M613T**	>10		This study
	2348T>C	10	V783A	>10		[99]
	2388C>A	10	N796K	>10		[99]

Novel mutations are in bold. * Mutations considered pathogenic by prediction Software, but excluded by segregation studies. ^§ ^Exons 34, 36 and 37 are specific to isoforms CD1, CD2 and CD3, respectively.

The pathogenicity of several exonic variants found in our patients and predicted to be pathogenic in previous studies and/or by prediction software was further investigated. The p.T1209A missense mutation in *CDH23 *has previously been reported in two affected families and considered as pathogenic [55,58]. However, we found it in five of 486 control alleles from French and Maghreban populations. The p.Y1719C missense mutation in *MYO7A *seems to represent a frequent sequence variant in the Moroccan population, with an estimated carrier frequency of 0.07 [100], and was observed in three out of 306 control alleles. The p.R302H mutation in *MYO7A*, which affects a residue within a non-conserved region of the motor domain, was detected in one out of 494 control alleles. Moreover, two of five different *MYO7A *cDNA clones isolated from three independent libraries were found to encode a histidine residue at codon position 302 [101], which further argues in favor of a non-pathogenic sequence variant. The p.E3088K missense mutation in *USH2A*, previously described by Dreyer et al., was present in three out of 306 control alleles, which argues in favor of a non-pathogenic sequence variant [26,28]. The missense mutation p.I5126T in *USH2A *has been reported as likely pathogenic [87]. We found it in two USH1 patients, who in addition carried two pathogenic mutations in *MYO7A*. We detected it in two individuals from the French control population, suggesting that it is a non-pathogenic sequence variant. The p.L555V mutation in *USH2A *has been found in homozygous state in one Spanish patient, together with a biallelic splice site variant (c.1841-2A>G) [26]. Numerous, presumably neutral, isocoding and intronic variants were also observed (listed in Additional file 2**Table S1**).

Twenty-six pathogenic or presumably pathogenic mutations in *MYO7A *were found in 19 patients, specifically, eight nonsense mutations, one nucleotide duplication, five nucleotide deletions, four splice site mutations, and eight missense mutations. Seven of these mutations had not been previously reported, including two nonsense mutations (p.K164X, p.C1198X), a nucleotide duplication (c.397dupC; p.H133fsX7), a nucleotide deletion c.3904delT (p.Y1302fsX97), a nucleotide substitution (c.1690+1G>A) predicted to alter the splice donor site of intron 14, and two missense mutations (p.K268R and p.P1220L) that change amino acid residues located in the motor head and the first MyTH4 domain of the myosin VIIa tail, respectively (Tables 2, 3 and Figure 1).

Three distinct pathogenic or presumably pathogenic mutations in *USH1C *were detected in three patients, specifically, a nucleotide duplication (c.238_239dupC; p.R80fsX69) already reported in several patients [3,4,27,53], a known missense mutation (p.R103H) affecting an amino acid residue located in the PDZ1 domain of the protein [27], and a novel missense mutation (p.R357W), predicted to affect the first coiled-coil domain of the protein. These mutations are expected to affect the three classes of harmonin isoforms (Tables 2, 3, Figure 1) [4].

Eight pathogenic or presumably pathogenic mutations in *CDH23 *were found in six patients, specifically, a previously reported mutation that affects splicing (c.6050-9G>A) [54], a novel nucleotide deletion (c.6404_6405delAG; p.E2135fsX31), and six missense mutations [55,56], four of which (p.R1189W, p.R1379P, p.D2639G, and p.R3043W) had not been previously reported. They affect amino acid residues located in the 11^th^, 13^th ^and 25^th ^cadherin repeat and the extracellular region adjacent to the transmembrane domain (3065-3085), respectively (Tables 2, 3 Figure 1). Intriguingly, the p.R1060W mutation, which affects a residue in the 10^th ^cadherin repeat that belongs to a canonical motif (DRE) predicted to bind Ca^2+ ^[57], has previously been reported in an isolated form of deafness, DFNB12 (cited in Astuto et al. [55]).

Two pathogenic or presumably pathogenic mutations in *PCDH15*, specifically, a nonsense mutation (p.R991X) [27] and a novel missense mutation (p.R1273S), were found in two patients. The missense mutation affects an amino acid residue located immediately after the 11^th ^cadherin repeat (Tables 2, 3, Figure 1). The large genomic rearrangement in *PCDH15 *previously reported by Le Guedard et al. [44] was not detected in this group of patients.

Three pathogenic or presumably pathogenic mutations in *USH1G *were found in three patients, specifically, an already reported nonsense mutation (p.W38X) [58], a novel nucleotide duplication (c.84dupC; p.D29fsX29), and a novel sequence variant (c.46C>G; p.L16V). This variant was absent from the control DNAs (0/666 alleles) and, according to the prediction software programs (NetGene2 and ESE finder), should create a splice donor site resulting in a premature stop codon at codon position 17 (Tables 2, 3; Figure 1).

Twenty-five pathogenic or presumably pathogenic mutations in *USH2A *were found in 17 patients including three USH3 patients, specifically, five nonsense mutations, one nucleotide duplication, six nucleotide deletions [59], two splice site mutations, 10 missense mutations, and one isocoding variation possibly creating a splice donor site (Tables 2, 3). All these mutations affect the extracellular region of usherin (Figure 1). Nine mutations had not been previously reported, specifically, five frame-shifting deletions (c.4030_4037delATGGCTGG/p.M1344fsX42, c.5189_5199delATATGTTTCAT/p.Y1730fsX6, c.7522delT/p.R2509fsX19, c.8970_8971delCA/p.T2991fsX61, and c.12381_12382delCT/p.Y4128fsX24), one splice acceptor site mutation (c.10586-1G>C) that is expected to result in exon 54 skipping and premature termination of the protein, and three missense mutations (p.C575Y, p.G1301V, p.C3307W) that affect amino acid residues located in the 14^th ^fibronectin type III domain and the trideca-di-cysteine domain (residue 3192 to 3371) between the 18^th ^and the 19^th ^fibronectin type III domains (Figure 1). Notably, the isocoding mutation (c.949C>A; p.R317R) has been predicted to be pathogenic by Pennings [60] and considered as nonpathogenic by Dreyer [28]. Segregation analysis in our family was compatible with a pathogenic effect of this mutation (Additional file 1 Figure S4).

Eleven pathogenic or presumably pathogenic mutations in *VLGR1 *were detected in eight patients including two USH3 patients. All were novel mutations, specifically, a nonsense mutation (p.E4321X), a nucleotide duplication (c.1563dupT; p.P552fsX8), four nucleotide deletions (c.333_334delTT/p.F112fsX29, c.2258_2270delAAGTGCTGAAATC/p.Q753fsX8, c.12552_12553delGG/p.E4186fsX17), and c.17668_17669delAT/p.M5890fsX10), and five missense mutations (p.D1944N, p.H3399P, p.D4707Y, p.N4885S, p.Q5459H) that all affect amino acid residues located in the large extracellular region of the protein, between the 13^th ^and 14^th ^β-Calx domains, in the 4^th ^Epilepsy Associated Repeat domain, in the 32^nd ^β-Calx domain, between the 32^nd ^and 33^rd ^β-Calx domains, and in the 35^th ^β-Calx domain, respectively (Tables 2, 3, Figure 1).

Three pathogenic or presumably pathogenic mutations in *WHRN *were detected in three patients including one USH3 patient, specifically, a novel deletion (c.737delC; p.P246fsX13), and two novel missense mutations (p.S11R and p.R379W) that affect amino acid residues located in the N-terminal Ala/Gly/Ser-rich stretch (aa 9-31) and immediately after the PDZ2 domain, respectively (Tables 2, 3, Figure 1). Notably, these missense mutations only affect the longer whirlin isoform [19], which is a component of the ankle link molecular complex together with VLGR1 and usherin [40,41].

No mutations in *USH3A *were detected in our series of USH patients.

### Transmission modes: evidence for digenic/oligogenic inheritance in some patients

We found mutations in 49 out of 54 (91%) USH patients, specifically, in 24 out of 27 (89%) USH1 patients, 19 out of 21 (90%) USH2 patients, and all six (100%) USH3 patients (see Table 1). Mutations in *MYO7A*, *USH1C*, *CDH23, PCDH15*, and *USH1G*, were found in 55%, 7%, 7%, 7%, and 4% of the USH1 cases, respectively. Mutations were detected on both alleles in 21 USH1 patients (including the six consanguineous families), and on one allele in the remaining three USH1 patients. Moreover, one of these patients (U3) harboured monoallelic, presumably pathogenic mutations in two different USH1 genes (see below). Mutations in *USH2A*, *VLGR1 *and *WHRN *were found in 57%, 19% and 9.5% of the USH2 cases, respectively. Notably, one USH2 patient (P0486) carried biallelic mutations in *MYO7A*. Mutations were detected on both alleles in 15 USH2 patients (including a consanguineous family), and on one allele in the remaining four USH2 patients. Finally, as regards the USH3 patients, biallelic mutations in *USH2A *and monoallelic mutations in *VLGR1 *or *WHRN *were found in three patients, two patients, and one patient, respectively.

**Figure 2:**
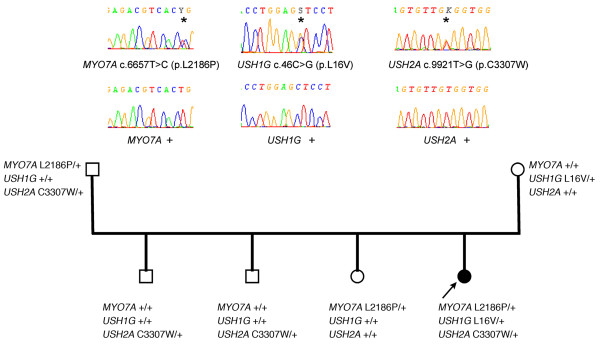
**Segregation of the mutations in *MYO7A*, *USH1G *and *USH2A *in family U3**. Arrow indicates the deaf proband.

One USH1 and two USH2 patients were heterozygotes for mutations in two or three USH genes, suggesting a possible digenic/oligogenic inheritance of the syndrome. In the USH2 patients, however, segregation analysis did not support digenic inheritance. Patient P0418 carries a nonsense mutation in *USH2A *(p.S5030X) and a missense mutation in *MYO7A *(p.K268R), but his brother, who is also clinically affected, does not carry the *MYO7A *mutation. Patient P0432 has a c.4030_4037delATGGCTGG (p.M1344fsX42) mutation in *USH2A *and a missense mutation in *CDH23 *(p.R1189W), but his father, who has neither deafness nor retinitis pigmentosa, also carries these two mutations, and his clinically affected sister does not carry the mutation in *CDH23*. In the USH1 patient, we found three presumably pathogenic mutations in *MYO7A *(c.6657T>C), *USH1G *(c.46C>G; p.L16V) and *USH2A *(c.9921T>G). Her father carries the mutations in *MYO7A *and *USH2A *without displaying symptoms of the disease, whilst her unaffected mother carries the mutation in *USH1G*. The mutations in *MYO7A*, *USH1G *and *USH2A *were not found in 666 control alleles. Of the four siblings, the affected girl is the only one who carries the mutations in *MYO7A *and *USH1G*, and, all the more, the mutations in the three genes (Figure 2). Therefore, a combination of monoallelic mutations in three USH genes may be responsible for the disease in this patient.

**Figure 3:**
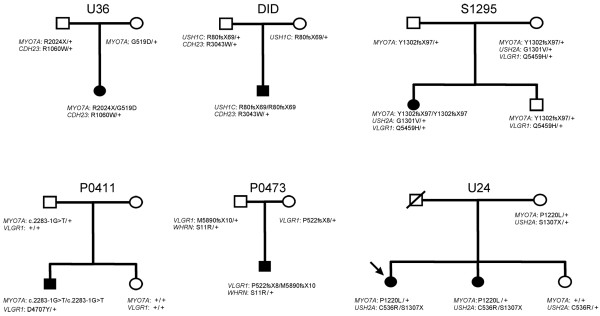
**Genetic evidence for presumably pathogenic mutations in more than one USH gene in six families**. The index case in family U24 is indicated by an arrow.

Seven patients out of 54 (13%) carried two presumably pathogenic mutations in an USH gene, plus one or two additional mutations in another USH gene. Taking into account only the 39 patients for whom biallelic mutations have been identified, 18% (7 out of 39) carry additional mutations. Specifically, five USH1 patients carried biallelic mutations in an USH1 gene plus one or two additional mutations in another USH1 (three patients) or USH2 (two patients) gene, and two USH2 patients carried biallelic mutations in USH2 genes plus one additional, presumably pathogenic mutation in an USH1 or an USH2 gene (Table 1). Parents and siblings available in six out of seven families indeed showed that the two mutations present in the same gene originated from one parent each (Figure 3). The mutations found in the genes that were mutated on both alleles in the patients consist of two nonsense mutations, five nucleotide deletions, one splice site mutation, and three missense mutations. The eight additional mutations found in these patients were amino acid substitutions that were predicted "probably damaging" and "not tolerated" by Polyphen and SIFT program, respectively. One of these mutations, p.R1060W in *CDH23*, has already been reported in USH patients [55].

## Discussion

The major goal of the study was to design a powerful and reliable strategy for molecular diagnosis of USH. For that purpose, some essential, so far missing information was gathered by: i) comparing the strategy for mutation detection currently in use with the here developed USH exome sequencing (including splice sites), ii) determination of whether the phenotype can restrict the mutation screening to the USH genes corresponding to the clinical subtype in a given patient, and iii) defining the possible existence of digenic/oligogenic inheritance of the disease in some patients.

We found mutations in eight of the currently known nine USH genes, in 49 out of 54 (91%) patients (Table 1). Two or more mutations were identified in 41 patients, including 39 patients (72%) with biallelic mutations, and one mutation was found in the remaining seven patients (13%), that is a total of 81 different mutations. Current diagnostic strategies use a genotyping microarray based on the arrayed primer extension method [24]. Were the international USH genotyping microarray used to identify the mutations, only 30 out of the 81 mutations (37%) would have been possibly detected because of the high prevalence of novel mutations, whatever the USH clinical type. Only 9 mutations previously reported as recurrent were detected in our series of patients (i.e. 11% of the mutations), specifically, c.1996C>T, c.223delG, c.1556G>A, c.494C>T, c.3719G>A and c.5749G>T in *MYO7A*, c.238_239dupC in *USH1C*, and c.2299delG and c.10712C>T in *USH2A*. Therefore, in the process of designing any strategy for USH molecular diagnosis, taking into account the high prevalence of novel mutations appears to be of major importance.

Previous mutation research studies performed in patients referred to medical genetic clinics showed high proportions of mutations for *MYO7A*, *CDH23 *and *PCDH15 *in USH1 patients [27], specifically, 29%-55% for *MYO7A *[61-64], 19%-35% for *CDH23 *[58], 11%-15% for *PCDH15 *[65], and for *USH2A *in USH2 patients [28,60,66], whereas the implication of *VLGR1 *and *WHRN *in the latter was minor [11,12]. The present analysis confirms these results by showing a major implication of *MYO7A *in USH1 (55% of the cases), and of *USH2A *in USH2 (62% of the cases).

Surprisingly, mutations were found in genes that did not fit the clinically diagnosed USH type. None of the six patients diagnosed as USH3 on the basis of the postlingual onset and progressive nature of the deafness, and the absence of vestibular dysfunction (see Subjects and methods) carried a mutation in *USH3A*. Yet, mutations in USH2 genes were present in all of them, and with a gene distribution similar to that observed in USH2 patients. This finding, which concerns six out of 24 patients carrying mutations in USH2 genes, calls for a revision of the USH2 clinical features. Along the same line, one patient diagnosed as USH2, because he did not have a vestibular dysfunction, carried biallelic missense mutations in an USH1 gene, *MYO7A*. The two mutations (p.A457V and p. K269del) affect amino acid residues located in the motor head of myosin VIIa, and have previously been reported in USH1 patients [63]. They may preserve a residual activity of the protein, thus causing less severe hearing, balance and visual impairments. Alternatively, one of these mutations or both might be deleterious for the myosin VIIa activity associated with the ankle-link protein complex that underlies the USH2 phenotype [40], but not with the transient hair bundle lateral-link and tip-link molecular complexes that are involved in USH1 pathogenesis. These phenotype/genotype discrepancies further argue in favor of a comprehensive mutation screening procedure that includes genes seemingly inconsistent with the clinical classification of USH currently in use.

Notably, our study has revealed one case of likely oligogenic inheritance for USH1, involving *MYO7A *and *USH1G*, and possibly *USH2A*. Three cases of digenic inheritance of USH1 have been reported so far [42], all caused by mutations in *CDH23 *and *PCDH15*, in agreement with the contribution of cadherin-23 and protocadherin-15 to the hair bundle transient lateral links and tip-links [31,32,36,67-69]. The pathogenicity of the p.T1209A mutation in *CDH23 *[18,55] is, however, questionable since we found it in five alleles from the control population. The c.5601delAAC mutation in *PCDH15*, leading to an in frame-deletion of a threonine residue (p.T1868del) [42] within the intracellular domain of the protocadherin-15 CD1 isoform, also warrants a special mention. Three protocadherin-15 isoforms (CD1-3) that differ in their intracytoplasmic regions have been reported [69]. Already two presumably pathogenic mutations (p.M1853L and p.T1868del) [42,70] have been found in exon 34 that is specific for CD1. Incidentally, the p.T1868del mutation was not only involved in USH1, but has also been found, in homozygous state, in a deaf patient presenting with vestibular arreflexia and without retinitis pigmentosa (C. Bonnet, unpublished). The CD2 isoform(s) of protocadherin-15 make(s) the transient kinociliary links [71], whereas the protocadherin-15 isoforms that make transient interstereocilia links and the tip-links are still unknown. The mutations in exon 34, however, point to an essential biological role of CD1, or of an as yet uncharacterized protocadherin-15 isoform that contains the amino acid sequence encoded by this exon, in the hair cells.

**Figure 4:**
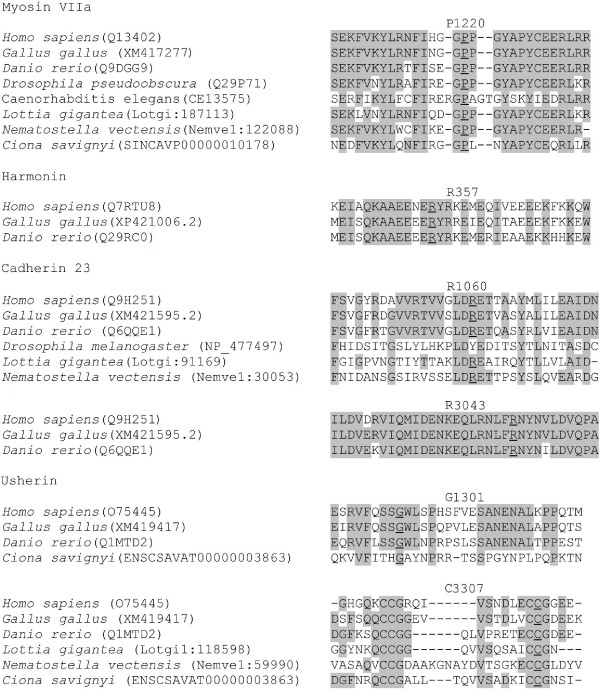
**Interspecies conservation of amino acid residues mutated in patients carrying presumably pathogenic mutations in several USH genes**. Representative stretches of amino acid sequences from each of the USH proteins from various species were aligned, and identical residues highlighted with shading. Residues involved in missense mutations are underlined. Protein ID accession numbers are indicated in parentheses. Orthologs of *MYO7A*, *USH2A *and *WHRN *are present in the cnidarian *Ciona savignyi*; they encode proteins that have 53.5%, 36.5%, and 24.7% (whirlin short isoform) of sequence identity with the human proteins, respectively. Notably, the P1220 residue of myosin VIIa, and the G1301 and C3307 residues of usherin, which are involved in the USH patients' missense mutations, are conserved in *C. savignyi*. Incidentally, all the new *USH2A *missense mutations detected in our series of patients affect residues that are also conserved in this species.

Therefore, even though non-monogenic inheritance of USH appears to be rare, it has to be taken into consideration in the molecular diagnosis strategy. In addition, ten patients had presumably pathogenic mutations in two different USH genes. Seven of them had biallelic mutations in one gene, and carried an additional mutation in a second and, for one of them, a third USH gene. None of these additional mutations were nonsense or frame-shifting mutations, but the conservation of the corresponding amino acid residues in the orthologous genes (*ush2a*, *myo7a*, *whrn*) of *Ciona savignyi *[72], a cnidarian which is evolutionary distant of about 520 million years from man [73], argues in favor of their pathogenicity (Figure 4). Notably, these mutations were not found in 402 to 666 control alleles from populations of matched geographic origin. A substantial proportion of USH patients thus carry a third, presumably pathogenic mutation which, in some cases, may contribute to worsen the sensory defects resulting from missense mutations present in the "primary" USH gene.

Finally, no mutations were detected in five patients, specifically three USH1 and two USH2 patients. In patient S1823 (USH1), born from consanguineous parents, involvement of any of the nine currently known USH genes could be excluded by segregation analysis of polymorphic markers at the corresponding loci (data not shown). In the four remaining patients, the undetected mutations might still be located in the unexplored promoter regions or intragenic regulatory sequences of these genes, but may also be located in other, still unknown USH genes, as in patient S1823. Indeed, a new locus, USH1H, at chromosome 15q22-23 [74], and three candidate regions for new USH2 genes (2q32, 4q26 and 15q22-23) have been reported [75].

## Conclusion

Direct exon sequencing of a set of specific disease genes is a reliable, easy set-up method, which remains less expensive than full exome sequencing in the patients. Based on the high prevalence of private mutations both in USH1 and USH2 patients, the substantial number of cases displaying genotype/phenotype discrepancy, and the presence of additional, presumably pathogenic mutations in a number of patients, we conclude that exon sequencing (including flanking splice sites) of all currently known USH genes is required for proper molecular diagnosis in every USH patient, both in the context of genetic counseling and in the perspective of retinal and cochlear gene therapy. The activity of the USH gene carrying biallelic mutations may indeed turn out to be only partly restored by gene therapy, and the presence of a third mutation in another USH gene may then critically impact on the benefits of the gene therapy. Moreover, as *PDZD7 *[76] has recently been reported to modify the phenotype in patients carrying mutations in *USH2A *or *VLGR1 *[77], future studies should also take into account modifier genes in the USH exome sequencing strategy.

## List of abbreviations

APEX: Arrayed Primer EXtension; CDH23: Cadherin 23; DNA: DeoxyriboNucleic Acid; ESE: Exonic Splicing Enhancers; MYO7A: Myosin VIIa; PCR: Polymerase Chain Reaction; PCDH15: Protocadherin 15; SIFT: Sorting Intolerant From Tolerant; USH: Usher syndrome; USH1: USH type I; USH2: USH type II; USH3: USH type III; VLGR1: Very Large G protein-coupled Receptor; WHRN: Whirlin.

## Competing interests

The authors declare that they have no competing interests.

## Authors' contributions

CB and MG contributed equally to this work. FD and CP conceived of the study and participated in its design and coordination. CB and MG carried out the molecular genetic study and analysed the data. SM, BD, CV, HD, MME, AD, CC, JV, BM, DB, JD, CT, AD, CF, TM, DL, FD, VDG, MFTO, SS, AMF, PC, GC contributed to clinical and genetic evaluation of the patients. DZ, MD, DF, MP, MNG, DW, ML participated in the study of the control population. JW provided DNA sequencing facilities. CB, JPH, FD, CP wrote the manuscript. MG, SM, DW, AEA, LJ, JL, JAS participated in manuscript writing. All authors have read and approved the final manuscript.

## Supplementary Material

Additional file 1 **Figure S1: Sequence alignment of amino acid residues mutated in patients carrying missense mutations in USH1 genes**. Representative stretches of amino acid sequences from each of the USH1 proteins in various species were aligned. Identical residues are highlighted with shading. Residues involved in missense mutations are underlined. **Figure S2: Sequence alignment of amino acid residues mutated in patients carrying missense mutations in USH2 genes**. Representative stretches of amino acid sequences from each of the USH2 proteins in various species were aligned. Identical residues are highlighted with shading. Residues involved in missense mutations are underlined. Orthologs of *VLGR1 *are not present in the genomes of invertebrates such as *C. elegans *and drosophila. **Figure S3: Missense mutations possibly creating or disrupting a splice site**. Representative stretches of amino acid sequences from each of the USH proteins in various species were aligned. Identical residues are highlighted with shading. Residues involved in missense mutations are underlined. Triangles indicate splice sites. Scores for splice sites are obtained by NetGene2 software program. Possible new splice sites are in bold. **Figure S4: Segregation analysis of the *USH2A *mutations in family U51**. Click here for file

Additional file 2 **Table S1**. Presumably neutral, isocoding and intronic variants in USH genes. Click here for file
