# Supplementary material for: Complete exon sequencing of all known Usher syndrome genes greatly improves molecular diagnosis
Source: Orphanet J Rare Dis. 2011 May 11;6:21. doi: 10.1186/1750-1172-6-21 (PMC3125325; doi:10.1186/1750-1172-6-21)
Supplement: Additional file 1 — Figure S1: Sequence alignment of amino acid residues mutated in patients carrying missense mutations in USH1 genes. Representative stretches of amino acid sequences from each of the USH1 proteins in various species were aligned. Identical residues are highlighted with shading. Residues involved in missense mutations are underlined. Figure S2: Sequence alignment of amino acid residues mutated in patients carrying missense mutations in USH2 genes. Representative stretches of amino acid sequences from each of the USH2 proteins in various species were aligned. Identical residues are highlighted with shading. Residues involved in missense mutations are underlined. Orthologs of VLGR1 are not present in the genomes of invertebrates such as C. elegans and drosophila. Figure S3: Missense mutations possibly creating or disrupting a splice site. Representative stretches of amino acid sequences from each of the USH proteins in various species were aligned. Identical residues are highlighted with shading. Residues involved in missense mutations are underlined. Triangles indicate splice sites. Scores for splice sites are obtained by NetGene2 software program. Possible new splice sites are in bold. Figure S4: Segregation analysis of the USH2A mutations in family U51. [file 1750-1172-6-21-S1.PDF]

USH sequencing   Bonnet - Petit 2011

Figure S1

Myosin VIIA

	G163 and T165	K268	A457	R1240
Homo sapiens(Q13402)	ISGESGAGKTESTKLILQFL	NYHVFYCMLEGMSEDQKKKL	SFEQLCINFANEHLQQFFVR	APYCEERLRRTFVNGTRTQP
Gallus gallus (XM417277)	ISGESGAGKTESTKLILQFL	NYHVFYCMLRGMTMEQKKKL	SFEQLCINFANENLQQFFVR	APYCEERLRRTFANGTRTQP
Danio rerio(Q9DGG9)	ISGESGAGKTESTKLILQFL	NYHIFYCMLKGMTPDQKKQL	SFEQLCINFANENLQQFFVR	APYCEERLRRTFVNGTRTQP
Drosophila pseudoobscura (Q29P71)	ISGESGAGKTESTKLILQYL	NYHVFYCILAGLSSEEKSRL	SFEQFCINYANENLQQFFVQ	APYCEERLKRTFNNGTRNQP
Caenorhabditis elegans(CE13575)	ISGESGAGKTESTKLVLQFL	NYHIFYCLLAGLSREEKSEL	SFEQLCINFANETLQQFFVH	SKYIEDRLRRTQVNGTRHQP
Lottia gigantea(Lotgi:187114)	ISGESGAGKTESTKLILQFL	NYHIFYCMLAGMNKEDKNKL	SFEQLCINYANENLQQFFVR	--------------------
Nematostella vectensis(Nemve1:30268)	ISGESGAGKTESTKFILQYL	NYHIFYRMLAGMTPKEKALL	SFEQLCINYANEHLQQFFVK	APYCEERLR-------RTQ-
Ciona savignyi(SINCAVP00000010178)	VSGESGAGKTESTKLVLQYL	NYHVFYCMLAGMAANKKQEL	SFEQLCINFANENLQQFFVR	APYCEQRLLRTFANGPRSQP

	R1883
Homo sapiens(Q13402)	LRNGSRKYPPHLVEVEAIQH
Gallus gallus (XM417277)	LRNGSRKYPPHLVEVEAIQH
Danio rerio(Q9DGG9)	LRNGSRKYPPHLVEVEAIQH
Drosophila pseudoobscura (Q29P71)	IRHGQRKYPPHQVEVEAIQH
Caenorhabditis elegans(CE13575)	AKGGSRKYPPHLVEVEAIQH
Lottia gigantea(Lotgi:187114)	LRNGPRKYPPHQVEVEAIQH
Nematostella vectensis(Nemve1:122088)	IRVGQRKYPPHIVEVEAIQH
Ciona savignyi(SINCAVP00000010178)	TTHGHRKYPPHLVEVEAIQH

Harmonin

	R103	R731
Danio rerio (Q29RC0)	LGLSVRGGLEFGSGLFISQI	SYEEETGEGFQKYVDDFDPH
Gallus gallus(XP421006.2)	LGISVRGGAEFSCGLFISQL	SFRSNKKQGFQKYVEDFDPH
Homo sapiens(Q7RTU8)	LGLSVRGGLEFGCGLFISHL	VYQTAFRQDFRKYEEGFDPY
Culex quinquefasciatus (B0X051)	FGFSIRGGLEYGTGFFVSAI	-PKPVRPPSYYFCPQGTGPA
Lottia gigantea(Lotgi:159301)	LGFAVRGGFEHGVGVFVSDV	--------------------
Nematostella vectensis(Nemve1:95751)	FGFCMRGGSEHGVGLYVSSI	--------------------

Cadherin 23

	H755	R1189	R1379	A3317
Homo sapiens(Q9H251)	DGGVGHNQKTGIATVNITLL	LGPMR---SSVRVIVYVEDI	GVITVQGLVDREKG--DFYT	ITAGNRERAFFINATTGIVT
Gallus gallus(XM421595.2)	DGGVGHHQKTGIAMVNITLL	HGPMR---SSVRVIVYVEDV	GVITVKGQVDREKG--DFYT	ITAGNKESAFYINSTTGIIY
Danio rerio (Q6QQE1)	DGGVGPQQKTGIATVNITVL	LGSMR---STVRVIVYVEDV	GRITVTGLLDRERG--DSYT	ITAGNSDGAFYINETSGVVQ
Drosophila melanogaster (NP_477497)	--------------------	QPSERQLSTEKLVTVIVEDI	GILSTAAELDREAGPEEYEV	IVLGDQNHDFSISEDTGVVR
Lottia gigantea(Lotgi:91169)	DQGIPSLSSTTTINIRVLDV	--------------------	--------------------	ITGGDSEKYFTIGKTTGAIT
Ciona savignyi(SINCSAVP00000012941)	--------------------	--------------------	--------------------	IDD--VDKTFSINKKSGQIY
Nematostella vectensis(Nemve1:30053)	--------------------	---------------------	--------------------	IISGNDEKRFKINSTSGVIM

	R2066	A2110	I2199	R2270
Homo sapiens(Q9H251)	ITILDDNDNRPTFSPATLTV	LVYRIEAGAQDRFLIHLVTG	VLESAEPGTVIANITAIDHD	SVIDNASDLPERSVSVPNAK
Gallus gallus(XM421595.2)	VTILDDNDNRPVFQPVSITA	LDYRIEGGGQDRFLIDATTG	VPESATPGTVVAEVTAIDRD	SVHDNASEVTDRSVSVPNAK
Danio rerio (Q6QQE1)	ITILDVNDNPPVFNPSSFTV	LQYRLDSGAQDRFIVDALSG	VNESTPPGVVVATLAAEDPD	SVHDNASDVIEMSVSVPNAK
Drosophila melanogaster (NP_477497)	VEVQDINDNDPVFELQSYHA	LRFNLLGEHMHRFHIDSETG	VPERISKGEFVFGARALDLD	VSATSPKKGLVGKIRYAIAG
Ciona savignyi(SINCSAVP00000012941)	VTVKDLNDNPPRFASRRVRT	LFYTIVSGAHDRFVVDRETG	--------------------	FIVDDVDQGLNCDVTYEIGG

	T2290	D2639	D3017	A3317
Homo sapiens(Q9H251)	VNVLDVNDNTPQFKPFGITY	VYATDKDEGLNGAVRYSFL	LLIHVVNRDTNRILDVDRVI	QKGLGRSLETLTAAEATAFE
Gallus gallus(XM421595.2)	VNVLDVNDNTPRFRPFGVTY	VYATDKDEGLNGAVLYSLL	LLIHVVNRETNRILDVERVI	QKVLGRSLETLTADCG-AYD
Danio rerio (Q6QQE1)	INVLDVNDNAPRFRPFGVAN	VFATDEDEGVNGEVRYSFL	VLIHVVNKQTNRILDVEKVI	QRALARSLEALHADGGLYAE
Drosophila melanogaster (NP_477497)	LITLNVTDANDNAPVMEQLI	AHAADKDSPKNAIIHYAFL	QLLMAVRLAKTSYQQPKYLI	QHRHTAPFVTRNQGGQPPPP
Ciona savignyi(SINCSAVP00000012941)	FIVDDVDQGLNCDVTYEIGG	-------------------	--------------------	--------------------

Protocadherin 15

	R1273
Homo sapiens (Q96QU1)	EKKIEDLTEILDRYVQEQIP
Gallus gallus (NP_001038119.1)	EQNKDQLIGILERYVQDQIP
Danio rerio (NP_001012500.1)	EENKEQLISILERYVQDQIP
Drosophila melanogaster (NP_733314.1)	PSDLRNYYTELEELLDKKTG

USH sequencing   Bonnet - Petit 2011

Figure S2

Usherin

	R303	T352	C536	C575
Homo sapiens (O75445)	RLHAQSHCRCPGSHPRVHPL	AHPLSFVNDNDVGTSWVSNV	CDTTSQPYRCLCSQESFTEG	YAFNCKPCQCNSHSKSCHYN
Gallus gallus (XM419417)	HLHTQSECRCPGSHPRVHPL	AHPLYYINDDDIGTTWISSV	CDTAMEPYRCLCIKESYTEG	HAYNCKPCQCYSHAVSCHYD
Danio rerio (Q1MTD2)	HLHSQSTCLCPASHPRVHPL	AHPLDYINDNDIGTTWISSI	CDTSVSPYRCACLPESHTHG	QAFNCRPCQCYGHASSCHYN
Lottia gigantea (Lotgi1 :161377)	EARIQTDCRCPPTHPRVKPQ	AHPLEFANDGDSNTVWIS--	CDMSTLPYKCNCLPASNTQG	KDYNCQLCQCYNHATSCYYN
Nematostella vectensis (Nemve1:1999[illegible]	KAYIATNCRCPPGYPKISTS	AHPSEYMTDADTTTYWQSAV	--------RCECDPSTFTQG	DPYPCVKCECNNHATACYYN
Ciona savignyi (ENSCSAVAT00000000386[illegible]	EVDIQPNCLCPEGFPRVHPL	AHPLAYINDDDSSTTWISSL	CNTTVNPYQCNCLEYSNTDG	SSYDCQQCECHQHALSCHYE

	G2752	V3003	T3571	T4809
Homo sapiens (O75445)	VQVTWKPPLIQNGDILSYEI	YWIFISVFNGVHSINSAGLH	EYSYQLKACTVAGCATSSKV	TNYSIGVEACTCFNCCSKGP
Gallus gallus (XM419417)	VKVSWKPPLIQNGEILNYEI	YQIFFQVFNGAHSINSEVVH	EYSYQLRACTVAGCADSSKV	TTYSIGVEACTCFNCCSKGP
Danio rerio (Q1MTD2)	VQVTWAAPLIPNGEIERYEI	YKLSLTVSNGPHNITSPEVN	EYIYQLRACTSAGCTDSTKV	TVYSVGVEVCTCFLCCSRGP
Lottia gigantea (Lotgi1 :161377)	--------------------	YNMSVEACTAGGCTKSSPIL	SYTYQIEAINAFGSVRSTPV	TKYNMSVEACTAGGCTKSSP
Nematostella vectensis (Nemve1:199904)	ATLNWTQPQTPNGPL-----	YEFFVNASNTVGYILSNIIS	FYTFAVNACTTVGCSRSGDA	TFYTFAVNACTTVGCSRSG-
Ciona savignyi (ENSCSAVAT00000003863)	ATIRWQSPTQENGPIIRYEI	YVFTLDVDNGAHNVTSSPVQ	EYEYTLRACTSVGCSSSIPI	TTYRVAVEMCTLGSMCTRGP

VLGR1

	Q345	V754	S1161	D1944
Homo sapiens (Q8WXG9)	HESHLKFQIVDDTIPEIAES	LSLDWVNVENQVLKSGYTSR	VSVSWQLFQNDSALQPGQEF	NITVEILPDEDPELDKAFSV
Mus musculus (NP_473394.3)	HESHLKFQIIDDLIPEIAES	LSLDRVSVDSDVLKSGYTSR	VSVAWQLFQN-ASLQPGQEF	SITVEILSDEEPELDKALTV
Danio rerio (ENSDARP00000020853)	HETDLRFSIRDDNIPEIAES	LTLDRTNVDNQILKPGFTSR	VTIYWQLFANDTPLEPYQEF	SIYLLISQDDVPELDKTFKV

	H3399	D3477 and Q3497	D4707	N4885
Homo sapiens (Q8WXG9)	FRWNGGSFVLHQKLPVRGVL	GDQNSIDIFIWEMGQSSFRYFQS	GFFTIADGESEASFDVHLLP	AVLPVSEKAANSQVGFESTA
Mus musculus (NP_473394.3)	FRWNGNNFAWHQTLPVRGVL	GNQNAIDIFVWEMGHSSLRYFQS	GFFTIADGESDASFDVHLLP	AILSVPEEAANSQVGFESAA
Danio rerio (ENSDARP00000020853)	FVWNRGSFFLHQSLELQDII	GLNPACEVFLWGSQQTVFQQTQS	GSVIILAGQRLAEIILTLLP	AVVSVPEVAANPVVGFASLA

	Q5459
Homo sapiens (Q8WXG9)	ELKPEKVPQVEVYFFVELYE
Mus musculus (NP_473394.3)	ELNPKKVHQVEMPFFVELYD
Danio rerio (ENSDARP00000020853)	EVQPDKNPEYEVWFLVEVYK

Whirlin

	L327
Homo sapiens (Q9P202)	GSEAEGSGLK-VGDQILEVN
Gallus gallus (ENSGALP00000010189)	GSEPTAGGMATVGDQILEVN
Danio rerio (XP_685827.3)	GSAAEYSGLK-VGDQILEVN
Caenorhabditis elegans (CE43560)	GGAAEEDGRLQEGDEIVEID
Lottia gigantea (Lotgi1:116246)	FSVAENAGIK-VGDQILDVN
Nematostella vectensis (Nemve1:95751)	HSAASRAGLK-CGDQIMDVN
Ciona savignyi (SINCSAVP00000007641)	GSAAENAGLK-AGDEILEVN

Figure S3

Splice site prediction
Score for splice sites (0.4 to 1)

Usherin

V218

Species	Sequence	Intron 3 / Exon 4	Allele	Score
Homo sapiens(O75445)	HLSVQVHQTKISFFINGVEK	tcttacaggTgcatcag	normal allele	0.92
Gallus gallus(XM419417)	HLSVQVHHSRISFFLNGWED	tcttacaggAgcatcag	mutated allele	0.76
Danio rerio (Q1MTD2)	HLALQIHETSVSLFLNGNEE			
Lottia gigantea (Lotgi1 :161377)	HLAIQVYRTTISFFLNGPRV			
Ciona savignyi (ENSCSAVAT00000003863)	HLAIQVHKTDISVFINGVES			

R317

Species	Sequence	Exon 6	Allele	Score
Homo sapiens(O75445)	HPRVHPLAQRYCIPNDAGDT	ggcacagCg gtactgcatt	normal allele	0
Gallus gallus(XM419417)	HPRVHPLIQRYCIPNGADDT	ggcacag **Ag** gtactgcatt	mutated allele	0.85
Danio rerio (Q1MTD2)	HPRVHPLVERYCIPNAANDT			
Ciona savignyi (ENSCSAVAT00000003863)	FPRVHPLEPRYCIPNGVPDT			

Myosin VIIA

L2186

Species	Sequence	Exon 48 / Intron 48	Allele	Score
Homo sapiens(Q13402)	KLLCETSLGYKMDDLLTSYI			
Gallus gallus (XM417277)	KLLCETSLGYKMDDLLTSYI			
Danio rerio(Q9DGG9)	KLLCETSLGYKMDDLLTSYI	gcgagacgtcacTggtgagggcgcat	normal allele	0.98
Drosophila pseudoobscura (Q29P71)	KLLCETSLGYKMDDLLTSYI	gcgagacgtcacCggtgagggcgcat	mutated allele	0.82
Caenorhabditis elegans(CE13575)	KLLLDTTVGYKMDDLLTSYI			
Lottia gigantea(Lotgi:187114)	KLLCETSLGYKMDDLLTSYI			
Nematostella vectensis(Nemve1:122088)	KLLCETSLGYKMDDLLTSYI			
Ciona savignyi(SINCAVP00000010178)	KLLCETSLGYKMDDLLTSYI			

Sans

L16

Species	Sequence	Exon 1	Allele	Score
Homo sapiens(Q495M9)	DQYHRAARDGYLELLKEATR			
Gallus gallus(E1C0X4)	DQYHRAARDGYLDLLKEATK			
Drosophila melanogaster (NP_610829)	DRFHKAAKDGLLDVLAAATR	ggctacctggagCtcctcaag	normal allele	0
Lottia gigantea(Lotgi:71589)	DRFIAAAQDGYLDLLQSATK	ggctacctggag **Gt** cctcaag	mutated allele	0.75
Nematostella vectensis(Nemve1:171827)	DLYFKAARDGNLNILKNATK			

Figure S4

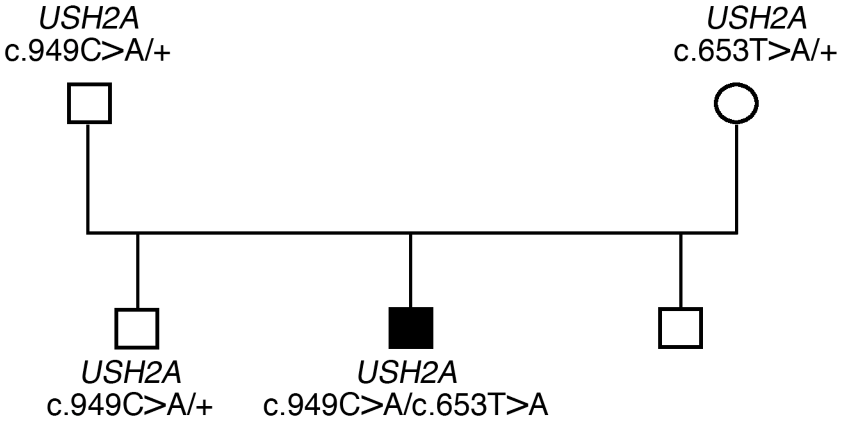

Segregation analysis of the USH2A mutations in family U51: The isocoding variation c.949C>A (p.R317R) has been predicted to be pathogenic by Pennings et al [1], and considered as non-pathogenic by Dreyer et al [2]. Segregation analysis of this variation in family U51 is compatible with a pathogenic mutation.

References

1. Pennings RJ, Te Brinke H, Weston MD, Claassen A, Orten DJ, Weekamp H, Van Aarem A, Huygen PL, Deutman AF, Hoefsloot LH, Cremers FP, Cremers CW, Kimberling WJ, Kremer H: ***USH2A* mutation analysis in 70 Dutch families with Usher syndrome type II.** *Hum Mutat* 2004, **24:**185.

2. Dreyer B, Brox V, Tranebjaerg L, Rosenberg T, Sadeghi AM, Moller C, Nilssen O: **Spectrum of *USH2A* mutations in Scandinavian patients with Usher syndrome type II.** *Hum Mutat* 2008, **29:**451.
