# Supplementary material for: Complete exon sequencing of all known Usher syndrome genes greatly improves molecular diagnosis
Source: Orphanet J Rare Dis. 2011 May 11;6:21. doi: 10.1186/1750-1172-6-21 (PMC3125325; doi:10.1186/1750-1172-6-21)
Supplement: Additional file 2 — Table S1. Presumably neutral, isocoding and intronic variants in USH genes. [file 1750-1172-6-21-S2.DOC]

**Additional table S1. Presumably neutral, isocoding and intronic variants in USH genes**

|  | **Nucleotide change** | **Exon** | **Frequency**  **in USH alleles (x/108)** | **References** |
| --- | --- | --- | --- | --- |
| ***MYO7A*** |  |  |  |  |
|  | **63C>T** | 3 | 2 | This study |
|  | **288G>A** | 5 | 5 | This study |
|  | **510G>A** | 6 | 3 | This study |
|  | 783T>C | 8 | >10 | [1] |
|  | **1006-24C>G** | 10 | 6 | This study |
|  | 1343+8G>A | 12 | >10 | [2] |
|  | 1343+32C>T | 12 | 2 | [2] |
|  | 1936-22G>A | 17 | >10 | [3] |
|  | 3375+33G>C | 26 | >10 | [2] |
|  | **3504+12delGAGGCGGGGACACCAGGGCCTG** | 28 | >10 | This study |
|  | 3924+12C>T | 30 | >10 | [2] |
|  | **4074C>T** | 31 | 1 | This study |
|  | **4323+35G>T** | 32 | >10 | This study |
|  | **4461C>T** | 34 | 1 | This study |
|  | **4755C>T** | 35 | >10 | This study |
|  | **5619G>A** | 40 | 6 | This study |
|  | **5715A<G** | 41 | >10 | This study |
|  | 5743-12T>C | 42 | >10 | [2] |
|  | **5857-7A>T** | 43 | >10 | This study |
|  | 6051+17T>A | 44 | >10 | [2] |
|  | 6052-11G>C | 45 | 1 | [4] |
|  | **6240C>T** | 46 | 6 | This study |
|  | **6318G>A** | 46 | >10 | This study |
|  | **6353+35G>A** | 46 | >10 | This study |
|  | 6439-31G>A | 48 | >10 | [2] |
|  | **6519C>T** | 48 | 1 | This study |
|  | 6558+16G>A | 48 | >10 | [2] |
|  | 6558+25A>G | 48 | 2 | [4] |
|  |  |  |  |  |
| ***USH1C*** |  |  |  |  |
|  | 37-45C>G | 2 | >10 | [5] |
|  | 105-16C>T | 3 | >10 | [6] |
|  | **249-49insGCGGGG** | 4 | >10 | This study |
|  | **522-48C>A** | 7 | >10 | This study |
|  | **580-27G>A** | 8 | >10 | This study |
|  | 819+10G>C | 10 | 1 | [7] |
|  | 1085+21C>G | 13 | >10 | [4] |
|  | **1086-44A>G** | 14 | >10 | This study |
|  | **1086-41G>A** | 14 | >10 | This study |
|  | **1086-12G>A** | 14 | 6 | This study |
|  | 1188A>G | 14 | >10 | [5] |
|  | **1321-34C>T** | 16 | >10 | This study |
|  | 1414-34G>A | 17 | >10 | [5] |
|  | **2340C>T** | 23 | >10 | This study |
|  | **2351-46G>A** | 24 | >10 | This study |
|  | **2490+12G>C** | 24 | >10 | This study |
|  | **2548-11T>C** | 26 | >10 | This study |
|  | 2656-47C>T | 27 | >10 | [7] |
|  |  |  |  |  |
| ***PCDH15*** |  |  |  |  |
|  | **157+3A>G** | 3 | 8 | This study |
|  | **243G>A** | 4 | 1 | This study |
|  | **319-32T>C** | 5 | >10 | This study |
|  | 475-3C>T | 6 | 1 | [4] |
|  | 546A>G | 6 | 1 | [8] |
|  | 960G>A | 9 | 2 | [1] |
|  | **1263T>C** | 11 | >10 | This study |
|  | **1797C>T** | 15 | 1 | This study |
|  | 2220+47T>C | 18 | >10 | [4] |
|  | 2221-34G>A | 19 | 4 | [4] |
|  | 2751+37C>T | 20 | >10 | [4] |
|  | **2751+43C>G** | 20 | 10 | This study |
|  | 3010-48G>A | 23 | >10 | [4] |
|  | **3717+35T>C** | 27 | >10 | This study |
|  | 3718-19C>A | 28 | 4 | [4] |
|  | 3984-20C>T | 30 | >10 | [4] |
|  | **4212-20C>T** | 32 | 1 | This study |
|  | **4581C>A** | 33 | >10 | This study |
|  | **4917C>T** | 37 | 1 | This study |
|  | **4950G>A** | 37 | 1 | This study |
|  |  |  |  |  |
| ***CDH23*** |  |  |  |  |
|  | -1C>T | 1 | >10 | [9] |
|  | 145+26C>T | 2 | 2 | [10] |
|  | **289-27C>A** | 4 | 1 | This study |
|  | 366T>C | 5 | >10 | [9] |
|  | 429+13G>A | 5 | >10 | [9] |
|  | 429+18T>C | 5 | 1 | [10] |
|  | 429+26A>G | 5 | >10 | [9] |
|  | 430-23A>C | 6 | 1 | [4] |
|  | 1038G>A | 10 | 1 | [9] |
|  | 1053C>T | 10 | 4 | [9] |
|  | **1089C>T** | 10 | 1 | This study |
|  | 1134+13A>G | 10 | 2 | [9] |
|  | 1753-43C>T | 16 | >10 | [10] |
|  | 2060-38G>T | 19 | >10 | [10] |
|  | 2060-19C>G | 19 | >10 | [10] |
|  | **2289+9G>A** | 20 | 1 | This study |
|  | 2316T>C | 21 | >10 | [9] |
|  | 2388T>C | 21 | >10 | [9] |
|  | 2397+26T>C | 21 | >10 | [9] |
|  | 2424G>A | 22 | 1 | [10] |
|  | 3009T>C | 25 | 3 | [9] |
|  | 3370-29G>A | 28 | 9 | [4] |
|  | 3370-46T>C | 28 | 5 | [4] |
|  | 3580-12C>T | 30 | 3 | [9] |
|  | **4068C>G** | 31 | 1 | This study |
|  | 4299T>A | 34 | 1 | [9] |
|  | 4488+32C>G | 35 | >10 | [9] |
|  | **4488+182 delG** | 35 | 1 | This study |
|  | 4509C>T | 36 | 1 | [10] |
|  | 4846-49T>C | 38 | >10 | [9] |
|  | 5187+44C>G | 39 | 3 | [9] |
|  | 5503-10A>G | 42 | >10 | [9] |
|  | 5503-44T>C | 42 | >10 | [9] |
|  | 5544C>T | 42 | 1 | [9] |
|  | 6852G>C | 49 | 1 | [10] |
|  | **6990G>T** | 49 | 1 | This study |
|  | 7055-16A>G | 50 | >10 | [10] |
|  | 7225-22C>T | 51 | 5 | [10] |
|  | 7572G>A | 53 | >10 | [9] |
|  | **8022G>A** | 55 | 1 | This study |
|  | **8308-3C>T** | 58 | 1 | This study |
|  | 8895C>T | 60 | >10 | [9] |
|  | 8980-14C>A | 61 | 1 | [9] |
|  | 9077+8G>A | 61 | >10 | [9] |
|  | 9319+11G>A | 64 | >10 | [10] |
|  | 9320-34C>T | 65 | 2 | [10] |
|  | 9873G>A | 69 | 7 | [9] |
|  |  |  |  |  |
| ***USH2A*** |  |  |  |  |
|  | **486-15C>T** | 3 | 2 | This study |
|  | 504A>G | 3 | >10 | [11] |
|  | 1419C>T | 8 | >10 | [11] |
|  | 1644+34A>C | 9 | 2 | [12] |
|  | 2167+17A>G | 12 | 1 | [13] |
|  | 3157+35G>A | 15 | >10 | [11] |
|  | **3811-8T>G** | 18 | >10 | This study |
|  | 4371G>A | 20 | 1 | [14] |
|  | 5013C>A | 25 | >10 | [15] |
|  | **6633+19A>G** | 36 | >10 | This study |
|  | 8558+40C>A | 42 | 2 | [13] |
|  | 8681+18A>G | 43 | 6 | [4] |
|  | **10062G>C** | 51 | 1 | This study |
|  | 10387-27T>C | 53 | >10 | [4] |
|  | 11389+9A>T | 58 | 2 | [4] |
|  | 11946G>A | 61 | >10 | [16] |
|  | 12612A>G | 63 | >10 | [17] |
|  | 12666A>G | 63 | >10 | [17] |
|  | 13191G>A | 63 | >10 | [15] |
|  | **14643-39A>G** | 67 | 1 | This study |
|  | 15298-24T>C | 71 | 3 | [4] |
|  | **15519+23G>A** | 71 | 3 | This study |
|  |  |  |  |  |
| ***VLGR1*** |  |  |  |  |
|  | **558+11T>A** | 5 | >10 | This study |
|  | **558+16A>T** | 5 | >10 | This study |
|  | **1086A>G** | 7 | 1 | This study |
|  | 2241-19G>T | 12 | >10 | [18] |
|  | 2367+8C>T | 12 | >10 | [18] |
|  | **2734-34C>G** | 15 | >10 | This study |
|  | **2898-41T>C** | 16 | >10 | This study |
|  | **3141A>G** | 17 | >10 | This study |
|  | **3279G>T** | 17 | >10 | This study |
|  | **4506C>T** | 21 | >10 | This study |
|  | **5304G>A** | 24 | 5 | This study |
|  | **5524-33T>C** | 27 | 1 | This study |
|  | **5524-36 delT** | 27 | >10 | This study |
|  | **5665-23T>C** | 28 | >10 | This study |
|  | **6949-10G>A** | 32 | >10 | This study |
|  | **7206G>A** | 33 | >10 | This study |
|  | **7945+6C>T** | 33 | 2 | This study |
|  | **7945+27C>A** | 33 | >10 | This study |
|  | **8034T>G** | 34 | 2 | This study |
|  | **8538T>G** | 37 | >10 | This study |
|  | **9213C>T** | 43 | 1 | This study |
|  | **9907-35A>C** | 47 | >10 | This study |
|  | **9927T>G** | 47 | >10 | This study |
|  | **10161+26G>T** | 48 | >10 | This study |
|  | **10769+9A>G** | 51 | 1 | This study |
|  | **10872A>G** | 52 | 4 | This study |
|  | **11472G>A** | 55 | 1 | This study |
|  | **11581-3insC** | 56 | >10 | This study |
|  | **11682C>T** | 56 | >10 | This study |
|  | **11874A>T** | 57 | 1 | This study |
|  | **12850-35A>T** | 64 | >10 | This study |
|  | **12850-31C>T** | 64 | 4 | This study |
|  | **12927G>A** | 64 | 4 | This study |
|  | **13599A>G** | 67 | >10 | This study |
|  | **13590C>T** | 67 | 4 | This study |
|  | **14649T>C** | 71 | 1 | This study |
|  | **16164A>G** | 75 | 4 | This study |
|  | **16248C>T** | 76 | 2 | This study |
|  | **18310-22A>G** | 87 | 3 | This study |
|  | **18609C>A** | 88 | >10 | This study |
|  | **18625-7T>C** | 89 | >10 | This study |
|  | **18735G>A** | 89 | 7 | This study |
|  |  |  |  |  |
| ***WHRN*** |  |  |  |  |
|  | **117G>A** | 1 | >10 | This study |
|  | **550-21A>G** | 4 | >10 | This study |
|  | **1204-127G>T** | 6 | 1 | This study |
|  | **1353T>C** | 6 | >10 | This study |
|  | **1416+22A>T** | 6 | >10 | This study |
|  | **1545G>A** | 7 | 5 | This study |
|  | **1627-12G>A** | 8 | >10 | This study |
|  | **2283C>T** | 10 | 9 | This study |
|  | **2721+40C>G** | 12 | 1 | This study |
|  |  |  |  |  |
| ***USH3A*** |  |  |  |  |
|  | 55A>T | 1 | >10 | [19] |

Novel mutations are in bold.

**References**

**1.** Hutchin T, Coy NN, Conlon H, Telford E, Bromelow K, Blaydon D, Taylor G, Coghill E, Brown S, Trembath R *et al*: **Assessment of the genetic causes of recessive childhood non-syndromic deafness in the UK - implications for genetic testing.** *Clin Genet* 2005, **68**(6):506-512**.**

**2.** Jaijo T, Aller E, Oltra S, Beneyto M, Najera C, Ayuso C, Baiget M, Carballo M, Antinolo G, Valverde D *et al*: **Mutation profile of the *MYO7A* gene in Spanish patients with Usher syndrome type I.** *Hum Mutat* 2006, **27**(3):290-291.

**3.** Jaijo T, Aller E, Beneyto M, Najera C, Graziano C, Turchetti D, Seri M, Ayuso C, Baiget M, Moreno F *et al*: ***MYO7A* mutation screening in Usher syndrome type I patients from diverse origins.** *J Med Genet* 2007, **44**(3):e71.

**4. The UMD central website.** http://www.umd.be

**5.** Zwaenepoel I, Verpy E, Blanchard S, Meins M, Apfelstedt-Sylla E, Gal A, Petit C: **Identification of three novel mutations in the USH1C gene and detection of thirty-one polymorphisms used for haplotype analysis.** *Hum Mutat* 2001, **17**(1):34-41.

**6.** Blaydon DC, Mueller RF, Hutchin TP, Leroy BP, Bhattacharya SS, Bird AC, Malcolm S, Bitner-Glindzicz M: **The contribution of *USH1C* mutations to syndromic and non-syndromic deafness in the UK.** *Clin Genet* 2003, **63**(4):303-307.

**7.** Aparisi MJ, Garcia-Garcia G, Jaijo T, Rodrigo R, Graziano C, Seri M, Simsek T, Simsek E, Bernal S, Baiget M *et al*: **Novel mutations in the USH1C gene in Usher syndrome patients.** *Mol Vis* 2010, **16**:2948-2954.

**8.** Ouyang XM, Yan D, Du LL, Hejtmancik JF, Jacobson SG, Nance WE, Li AR, Angeli S, Kaiser M, Newton V *et al*: **Characterization of Usher syndrome type I gene mutations in an Usher syndrome patient population.** *Hum Genet* 2005, **116**(4):292-299.

**9.** Astuto LM, Bork JM, Weston MD, Askew JW, Fields RR, Orten DJ, Ohliger SJ, Riazuddin S, Morell RJ, Khan S *et al*: ***CDH23* mutation and phenotype heterogeneity: a profile of 107 diverse families with Usher syndrome and nonsyndromic deafness.** *Am J Hum Genet* 2002, **71**(2):262-275.

**10.** Oshima A, Jaijo T, Aller E, Millan JM, Carney C, Usami S, Moller C, Kimberling WJ: **Mutation profile of the *CDH23* gene in 56 probands with Usher syndrome type I.** *Hum Mutat* 2008, **29**(6):E37-46.

**11.** Adato A, Weston MD, Berry A, Kimberling WJ, Bonne-Tamir A: **Three novel mutations and twelve polymorphisms identified in the USH2A gene in Israeli USH2 families.** *Hum Mutat* 2000, **15**(4):388.

**12.** Pennings RJ, Te Brinke H, Weston MD, Claassen A, Orten DJ, Weekamp H, Van Aarem A, Huygen PL, Deutman AF, Hoefsloot LH *et al*: ***USH2A* mutation analysis in 70 Dutch families with Usher syndrome type II.** *Hum Mutat* 2004, **24**(2):185.

**13.** Dreyer B, Brox V, Tranebjaerg L, Rosenberg T, Sadeghi AM, Moller C, Nilssen O: **Spectrum of *USH2A* mutations in Scandinavian patients with Usher syndrome type II.** *Hum Mutat* 2008, **29**(3):451.

**14.** Weston MD, Eudy JD, Fujita S, Yao S, Usami S, Cremers C, Greenberg J, Ramesar R, Martini A, Moller C *et al*: **Genomic structure and identification of novel mutations in usherin, the gene responsible for Usher syndrome type IIa.** *Am J Hum Genet* 2000, **66**(4):1199-1210.

**15.** Aller E, Jaijo T, Beneyto M, Najera C, Oltra S, Ayuso C, Baiget M, Carballo M, Antinolo G, Valverde D *et al*: **Identification of 14 novel mutations in the long isoform of USH2A in Spanish patients with Usher syndrome type II.** *J Med Genet* 2006, **43**(11):e55.

**16.** McGee TL, Seyedahmadi BJ, Sweeney MO, Dryja TP, Berson EL: **Novel mutations in the long isoform of the USH2A gene in patients with Usher syndrome type II or non-syndromic retinitis pigmentosa.** *J Med Genet* 2010, **47**(7):499-506.

**17.** Kaiserman N, Obolensky A, Banin E, Sharon D: **Novel *USH2A* mutations in Israeli patients with retinitis pigmentosa and Usher syndrome type 2.** *Arch Ophthalmol* 2007, **125**(2):219-224.

**18.** Deprez L, Claes LR, Claeys KG, Audenaert D, Van Dyck T, Goossens D, Van Paesschen W, Del-Favero J, Van Broeckhoven C, De Jonghe P: **Genome-wide linkage of febrile seizures and epilepsy to the FEB4 locus at 5q14.3-q23.1 and no *MASS1* mutation.** *Hum Genet* 2006, **118**(5):618-625.

**19.** Aller E, Jaijo T, Oltra S, Alio J, Galan F, Najera C, Beneyto M, Millan JM: **Mutation screening of USH3 gene (clarin-1) in Spanish patients with Usher syndrome: low prevalence and phenotypic variability.** *Clin Genet* 2004, **66**(6):525-529.
